# Absence of inferior vena cava in 14-year old boy associated with deep venous thrombosis and positive Mycoplasma pneumoniae serum antibodies- a case report

**DOI:** 10.1186/s12887-015-0357-0

**Published:** 2015-04-14

**Authors:** Boleslaw Kalicki, Monika Sadecka, Agata Wawrzyniak, Piotr Kozinski, Miroslaw Dziekiewicz, Anna Jung

**Affiliations:** Department of Pediatrics, Pediatric Nephrology and Allergy, Military Institute of Medicine, ul. Szaserow 128, 04-141 Warsaw, Poland; Department of Radiology, Military Institute of Medicine, ul. Szaserow 128, 04-141 Warsaw, Poland; Department of Vascular and Endovascular Surgery, Military Institute of Medicine, ul. Szaserow 128, 04-141 Warsaw, Poland

**Keywords:** Absence of inferior vena cava, Appendectomy, Deep venous thrombosis, Hypercoagulability, Low-molecular-weight heparin, Mycoplasma pneumoniae antibodies

## Abstract

**Background:**

Absence of the inferior vena cava is a rare vascular anomaly, which usually remains asymptomatic in childhood. It is recognized as the risk factor for deep venous thrombosis, since the collateral circulation does not provide adequate drainage of the lower limbs. Mycoplasma pneumoniae is a common cause of community-acquired pneumonia in school-aged children and adolescents. Mycoplasma pneumoniae infection might be associated with deep venous thrombosis but its pathophysiology remains unknown. According to previous reports, deep venous thrombosis due to Mycoplasma pneumoniae infection is associated with positive serum anticardiolipin antibodies. To our knowledge, we describe the first case of deep venous thrombosis associated with Mycoplasma pneumoniae serum antibodies indicating early stage of infection with negative anticardiolipin serum antibodies in adolescent with absence of inferior vena cava.

**Case presentation:**

14-year old boy was admitted to the pediatric unit few days after the appendectomy complaining with pain of the left hip that caused him unable to walk. The pain was accompanied with subfebrile temperature. After clinical examination and additional tests, the boy was diagnosed with a deep venous thrombosis. Computed tomography revealed absence of the vena cava inferior distally to the hepatic veins and varices of the collateral circulation in the pelvis. Anticardiolipin IgM and IgG antibodies and antinuclear antibodies were not detected. Additionally, the Mycoplasma pneumoniae antibodies in classes IgM, IgA and IgG were detected in serum as another risk factor of thrombosis. After the initial treatment with low-molecular-weight heparin in combination with clarithromycin the clinical condition of the patient improved. The patient became a candidate for life-long anticoagulation therapy.

**Conclusions:**

In this case Mycoplasma pneumoniae antibodies were associated with deep venous thrombosis in child with congenital absence of inferior vena cava. Uncommonly for deep venous thrombosis due to Mycoplasma pneumoniae infection, anticardiolipin antibodies were not detected in serum. It is important to remember in clinical practice that Mycoplasma pneumoniae affects coagulability and may trigger thrombosis, especially in the presence of other risk factors. The pathophysiology of this process remains unknown.

## Background

Congenital absence of the inferior vena cava (AIVC) is a rare vascular anomaly, often asymptomatic and identified serendipitously. Because AIVC is a defect that cannot be detected using b-mode USG, its prevalence is underestimated. The prevalence of AIVC has been estimated at 0.6-4% but some researches based on CT and/or MRI reported that AIVC may be present in 5–9.5% of young subjects with venous thrombosis. None of these studies evaluated AIVC prevalence in the general population [1,2]. Inferior vena cava (IVC) anomalies, including AIVC, are increasingly being recognized as the risk factors for deep vein thrombosis (DVT), since the collateral circulation does not provide adequate drainage of the lower limbs. Thrombosis associated with AIVC is often reported as a bilateral DVT that occurs in young adults, much younger than the mean age of DVT presentation [1]. Because the immature coagulation system is not promoting thrombosis, AIVC usually remains asymptomatic in children, manifesting in the early adults, especially in presence of thrombosis risk factors [3]. Some reports describe cases of DVT due to IVC anomalies in children and adolescents [4-7]. There is no standard management strategy established for patients with DVT due to AIVC. In most cases, life-long anticoagulation therapy could be indicated while there are some reports of recurrence of thrombosis after discontinuation of the treatment [8]. At least in one case, surgical correction with prosthetic venous bypass was necessary, when pharmacological treatment for stasis ulceration of lower limb, caused by AIVC, failed [9]. Therapeutic approach in children with DVT is different from the strategy in adults. In children with first DVT secondary to structural venous abnormalities either unfractionated heparin (UFH) or low-molecular-weight heparin (LMWH) are suggested for acute anticoagulant therapy and ongoing treatment [10,11]. M. pneumoniae is a common cause of community-acquired pneumonia in school-aged children and adolescents but its association with thrombosis is yet not well described. Previously reported extrapulmonary manifestations rarely applied to thrombosis and the pathophysiology of hypercoagulability in M. pneumoniae infection remains unknown. Most of the few reported cases of thrombosis applied to arterial location [12]. In several cases of M. pneumoniae infection, transient antiphospholipid antibodies (aPL), such as anticardiolipin antibodies (aCL), lupus anticoagulant and beta-2 microglobulin antibodies, have been reported, which might contribute to hypercoagulability [12-14].

In this article, we present a description of deep venous thrombosis associated with M.pneumoniae positive serum antibodies, indicating early infection, and negative aCL antibodies, in adolescent with congenital absence of IVC.

## Case presentation

**Figure 1:**
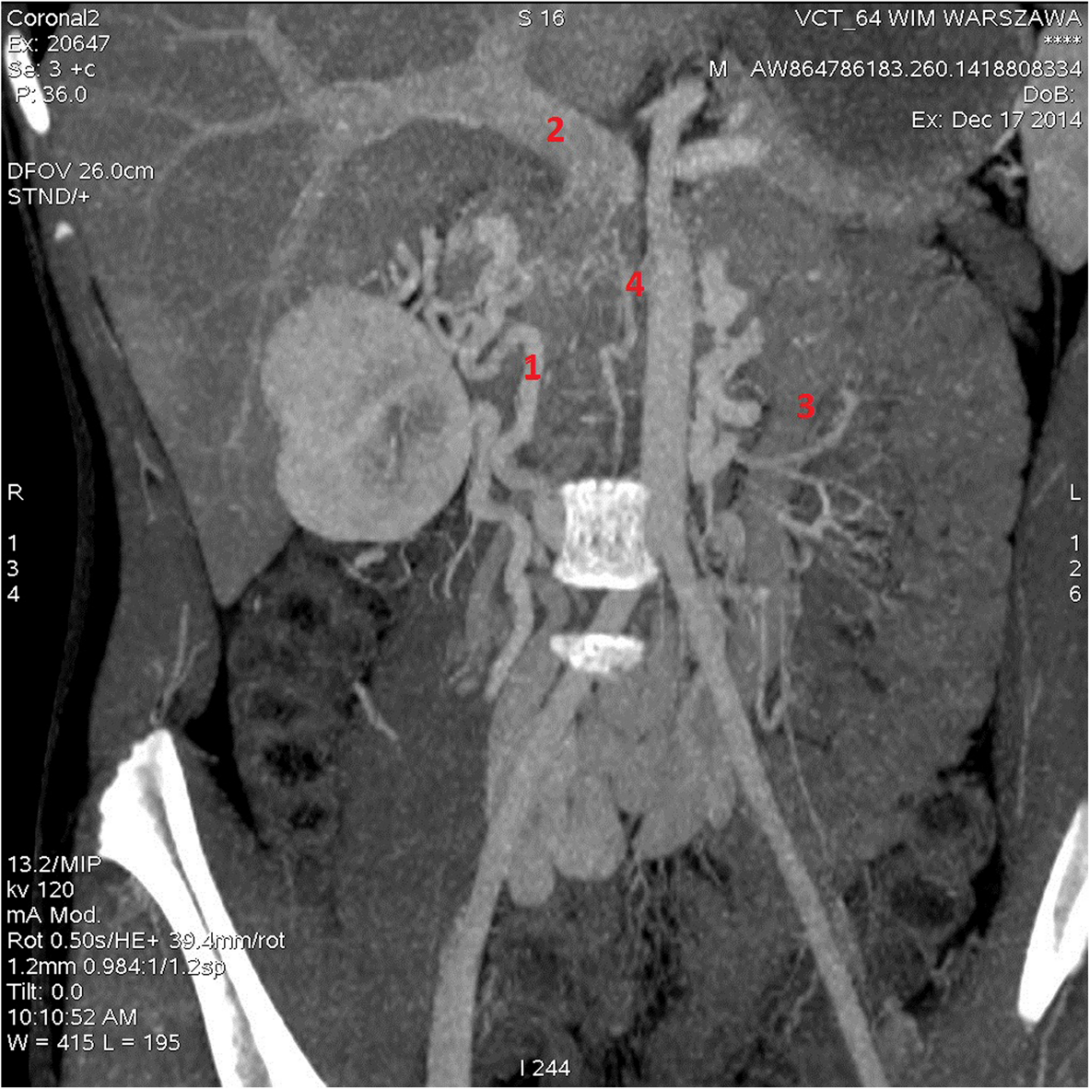
CT-angiograph of 14-year old boy with absence of inferior vena cava. Scan shows lack of contrast filling at the site of vena cava inferior. Numerous veins of collateral circulation within pelvis are varicosely dilated. Because of their atypical anatomy drainage of the renal veins could not be identified.1. Renal confluence, 2. Dilated portal vein, 3. Inferior mesenteric vein, 4. Lack of contrast filling at the site of inferior vena cava.

14-year old Caucasian boy was admitted to the pediatric unit complaining with severe pain of the left hip and internal thigh area that was exacerbated by compression and flexion of a hip. The patient was unable to walk and stand upright. Clinical examination revealed femoral artery pulse asymmetry with weaker left femoral pulse combined with asymmetrically increased circumference of the left lower limb greater by 1 cm at the levels of mid-thigh and mid-calf. Additionally, salmon-colored rush was spread on his trunk. 2 weeks before admission the patient underwent appendectomy with removal of purulent but not perforated appendix. Ultrasonography (USG) with the Doppler probe during postoperative period provided atypical image with difficulties in visualizing the inferior vena cava. The pain occurred 10 days after surgery and was constricting the movement of the left hip. Continuously aggravating pain was accompanied with subfebrile temperature (<38°C [100.4°F]) and elevated C-reactive protein (CRP) levels. The abdominal USG and roentgenograms of the hip showed no abnormalities, but amoxicillin treatment has been initiated. After 5 days, due to the constantly elevated CRP levels and temperature that raised up to 38°C [100.4°F], the patient was referred to the pediatric unit. At admission CRP level was in 8-fold reference range, D-dimer and fibrinogen concentrations were also elevated with values 326 μg/ml and 615 mg/dL, respectively. The ultrasonography showed deep venous thrombosis of left lower limb, vena cava inferior was not visualized. CT angiogram (Figure 1.) revealed the absence of inferior vena cava from common iliac veins connection to the confluence of hepatic veins. The patient had a short segment of inferior vena cava between the normal sized hepatic veins and the right atrium. The venous drainage from lower limbs and pelvis was found to be supplied by collateral circulation that includes varicosely dilated veins: azygos and hemiazygos, paraspinal and mesenteric. Additionally, the CT angiogram showed thrombus within left-sided popliteal vein, femoral vein, external and internal iliac veins and common iliac vein to the level of L4 vertebra. Further diagnostics were consequently performed. Anticardiolipin IgM and IgG antibodies and antinuclear antibodies were not detected. Tests for rheumatoid factor, anti-CCP antibodies and thrombophilia screening, which included genetic diagnostics for Leiden V factor, protein C and protein S, were also negative. The antithromobotic treatment has been started with 80 mg of enoxaparin s.c. twice daily with elevation and compression dressings for the full length of left lower limb. Control laboratory tests performed after 2 days of low molecular weight heparin (LMWH) treatment revealed that CRP, D-dimer and fibrinogen concentrations have lowered. Patient’s temperature was constantly elevated and did not decrease below 38°C [100.4°F]. Enzyme immunoassay (EIA) in serum detected Mycoplasma pneumoniae IgM, IgA and IgG antibodies with concentrations 1.258 S/CO, 25.183 EIU and 170.619 EIU, respectively. This pattern is typical for the early stage of infection. The antibiotic was changed to clarithromycin p.o. with the effect of normothermia after two days of treatment. The pain relieved, skin rash faded and after a couple of days both disappeared. The patient received clarithromycin in the recommended dose for 14 days. The initial antithrombotic treatment has been continued. The patient was advised to use fitted compression stocking, elevate leg at rest and avoid thrombogenic risk factors such as strenuous physical activity or prolonged immobilization. After 1 month, Mycoplasma pneumoniae antibodies in classes IgM, IgA and IgG were constantly being detected in serum and the concentrations indicated early infection. USG performed after 3 months of LMWH treatment showed reduction of initial thrombosis with recanalization of the left popliteal vein. The antithrombotic treatment has been continued with 80 mg of enoxaparin s.c. twice daily with subsequent USG control planned after following 3 months.

## Conclusions

AIVC is a rare vascular anomaly and the risk factor for deep venous thrombosis, that occurs predominantly in young adults [1,2]. There are only few descriptions of DVT due to AIVC presentation in children [4-7]. M. pneumoniae infection, popular among school children, might contribute to hypercoagulability and cause thrombosis itself. The pathophysiology of this process remains unknown, but transient aPL antibodies, such as aCL antibodies, are often detected in cases of DVT associated with M. pneumoniae infection [12-14]. In our patient, 14-year-old boy, many factors contributed to the unusual clinical presentation. The postoperative period was not connected with prolonged immobilization, but purulent appendix was a site of local inflammation before the surgery, that might have contributed to hypercoagulability. Developing M.pneumonie infection, in the presence of congenital AIVC, led to the lower limb DVT. Uncommonly for DVT due to M. pneumoniae infection, serum aCL antibodies were not detected.

To our knowledge, this is the first reported case of deep venous thrombosis associated with M.pneumoniae serum antibodies indicating early infection with negative aCL serum antibodies in adolescent with absence of inferior vena cava.

## Consent

Written informed consent was obtained from parents of the patient and the patient himself for publication of this Case report and any accompanying images. A copy of the written consent is available for review by the Editor of this journal.

## Abbreviations

aCL: Anticardiolipin antibodies
AIVC: Absence of the inferior vena cava
aPL: Antiphospholipid antibodies
CT: Computed topography
DVT: Deep vein thrombosis
IVC: Inferior vena cava
LMWH: Low-molecular-weight heparin
M pneumoniae: Mycoplasma pneumoniae
MRI: Magnetic resonance imaging
